# *Cylindropuntia cholla* Assisted Green Synthesis of Ag-Doped ZnO Nanoparticles for Methylene Blue Degradation

**DOI:** 10.3390/ma19081627

**Published:** 2026-04-18

**Authors:** Dillian Toledo Rodríguez, Guillermo Amaya Parra, Hugo A. Borbón Núñez, Franklin MuñozMuñoz, Priscy A. Luque Morales

**Affiliations:** Facultad de Ingeniería, Arquitectura y Diseño, Universidad Autónoma de Baja California, Ensenada C.P. 22860, BC, Mexico; dillian.toledo8@uabc.edu.mx (D.T.R.); hborbon@uabc.edu.mx (H.A.B.N.); franklin.muoz@uabc.edu.mx (F.M.)

**Keywords:** green synthesis, ZnO, doping, photocatalysis

## Abstract

Zinc oxide (ZnO) is a semiconductor with photocatalytic activity, although it presents limitations due to its band gap and the rapid recombination of the electron–hole pair; therefore, strategies such as doping have been explored. In this work, ZnO nanoparticles doped with 3% and 5% silver (Ag) were synthesized using a *Cylindropuntia cholla* root extract as a reducing and stabilizing agent. The structural, chemical, and optical properties of the synthesized nanoparticles were investigated using Fourier Transform Infrared Spectroscopy (FT-IR), X-ray Diffraction (XRD), Cathodoluminescence (CL), X-ray Photoelectron Spectroscopy (XPS), and Energy-Dispersive X-ray Spectroscopy (EDS). FT-IR shows that the nanoparticles have peaks between 400 cm^−1^ and 406 cm^−1^, attributed to the Zn–O bond. XRD characterization confirmed the formation of the wurtzite crystalline phase of ZnO, as well as the cubic phase of Ag. CL reveals two peaks: one attributed to the ultraviolet (UV) region and another in the visible region, which is associated with defects in the lattice. XPS and EDS confirm the presence of Zn, O, and Ag in the samples. The degradation of methylene blue was 90.9%, 96.4%, and 97.0% for ZnO, 3AgZnO, and 5AgZnO, respectively, demonstrating an improvement in dye degradation efficiency when doping ZnO nanoparticles with Ag.

## 1. Introduction

Water pollution is a worldwide issue that can originate from both natural processes and human activities [1]. Among the activities carried out by humans, the pharmaceutical, cosmetic, and textile industries stand out as major polluters, generating toxic and difficult-to-remove waste [2]. A wide variety of dyes are used in industrial processes, and they are difficult to degrade due to their complex structures and high stability; furthermore, their persistence can cause the coloration of water bodies, hindering the penetration of sunlight [3,4,5,6]. During industrial dye use, about 20% of the dye is lost and ends up in wastewater [7]. Due to their high solubility in water, dyes are difficult to remove through conventional treatment methods [8].

One of the most widely used dyes in the dyeing industry is methylene blue (MB). It is commonly used to dye silk, wool, cotton, and paper. MB is an aromatic heterocyclic basic dye with a molecular weight of 373.90 mol^−1^ and the molecular formula C_16_H_18_ClN_3_S [9]. It is highly soluble in water, forming a stable aqueous solution at room temperature [10]. Given the challenge of degrading dyes such as MB, there is growing interest in more sustainable and efficient technologies [11]. Nanotechnology offers alternatives through semiconducting materials with photocatalytic activity, capable of degrading organic compounds [12,13].

Zinc oxide (ZnO) is a semiconductor with a band gap of approximately 3.4 eV. ZnO nanoparticles have been used in various applications such as solar cells, gas sensors, photodetectors, anticancer agents, sunscreens, and catalysts due to their high biocompatibility, low toxicity, and low cost [14,15]. ZnO has three crystalline structures: zinc blende, cubic, and wurtzite (hexagonal). Under moderate temperature and pressure conditions, the wurtzite structure is thermodynamically stable, with lattice constants a = b = 0.32495 nm and c = 0.52069 nm. Under high-pressure conditions, the cubic structure is formed. In the hexagonal structure of ZnO, each O anion is bonded to four Zn cations [16].

ZnO presents certain limitations due to the rapid electron–hole recombination and because it can only be excited under ultraviolet light [17]. When ZnO is doped, different characteristic bands are generated depending on the type of dopant [18]. Metal dopants can act as electron donors or acceptors, while nonmetals generate a new valence band. The formation of intermediate bands reduces the energy required to excite the electron from the valence band to the dopant acceptor level [19,20,21]. Additionally, the incorporation of dopants has been widely reported to modify the electronic structure and promote the formation of reactive species, thereby enhancing catalytic performance in various systems [22].

Vallejo et al. (2020) demonstrated that 5% Ag doping of ZnO thin films, synthesized via a sol–gel method, significantly enhanced the degradation efficiency of MB, increasing it from 2.7% to 45.1% under visible light irradiation [23]. Additionally, Ag-decorated ZnO nanoparticles synthesized by co-precipitation have shown enhanced photocatalytic performance, achieving up to 95% degradation of organic dyes compared to lower efficiencies for pure ZnO. This improvement is mainly attributed to reduced electron–hole recombination and improved charge separation [24]. Overall, Ag doping of ZnO improves the photocatalytic degradation of dyes [25].

The synthesis of nanomaterials is a promising field; however, the process often involves the use of stabilizing and reducing agents that can be toxic and polluting. This has led to alternatives such as green synthesis, in which parts of plants, bacteria, fungi, or algae are used as reducing and stabilizing agents, providing an alternative to toxic compounds [26]. Studies have demonstrated the effectiveness of plant-mediated strategies for synthesizing Ag–ZnO nanomaterials. For instance, *Hylocereus costaricensis* extract has been successfully used for the synthesis of Ag/ZnO (bimetallic nanoparticles) [27]. Similarly, the use of goji berry extract enabled the synthesis of Ag@ZnO (nanocomposite) with enhanced photocatalytic performance, improving the degradation of MB. These approaches highlight the potential of natural resources to produce functional nanomaterials with enhanced properties while minimizing environmental impact [28].

*Cylindropuntia cholla* is a species belonging to the Cactaceae family. It is endemic to northwestern Mexico, mainly found in Baja California and Baja California Sur [29]. It is commonly known as cholla. In 2022, Reyes-Becerril et al. analyzed the chemical content of the cholla root using gas chromatography spectroscopy [30]. They identified 44 chemical compounds, with a high content of alditols such as glucitol (25.12%) and glycerol (9.04%). Studies have used commercial glycerol for the synthesis of gold (Au) nanoparticles, showing that the amount of glycerol employed is proportional to the nanoparticle size. Using glycerol, researchers successfully synthesized Au nanoparticles without the use of additional chemical agents [31,32].

Although extensive research has been conducted on ZnO and Ag-doped ZnO, no studies have reported the use of *Cylindropuntia cholla* root extract as a reducing and stabilizing agent. This root is rich in bioactive compounds that can influence nanoparticle formation during green synthesis. While Ag doping has been widely studied to enhance ZnO, the relationship between green synthesis conditions, material properties, and photocatalytic efficiency is not fully understood. In this context, this work aims to synthesize Ag-doped ZnO nanoparticles using *Cylindropuntia cholla* root extract and to evaluate the effect of Ag incorporation on their structural, morphological, and photocatalytic properties for MB degradation. This study contributes to the development of sustainable synthesis strategies while providing further insight into the role of Ag doping in improving photocatalytic performance.

## 2. Materials and Methods

### 2.1. Materials

The synthesis was carried out using zinc nitrate hexahydrate (Zn(NO_3_)_2_·6H_2_O, 99%, Framar, Niagara Falls, ON, Canada), silver nitrate (AgNO_3_, 99%, Sigma-Aldrich, St. Louis, MO, USA), deionized water (H_2_O), methylene blue, and *Cylindropuntia cholla* (cholla root) purchased from a local herbal store in Ensenada, Baja California, Mexico.

### 2.2. Preparation of the Extract

The cholla root was ground to a fine powder. A 2% (*w*/*v*) solution was prepared, stirred for 120 min, and subsequently heated in a water bath at 60 °C for 60 min. The extract was vacuum-filtered and stored for further use.

### 2.3. Synthesis

#### 2.3.1. ZnO Synthesis

A volume of 50 mL of the extract was mixed with 2 g of the zinc precursor (Zn(NO_3_)_2_·6H_2_O) for 120 min. The mixture underwent heat treatment at 60 °C for an overnight period. The resulting ZnO nanoparticles were calcined at 400 °C for 60 min.

#### 2.3.2. Synthesis of Ag-Doped ZnO

The same initial procedure was followed by mixing 50 mL of the extract with 2 g of Zn(NO_3_)_2_·6H_2_O for 60 min, followed by overnight thermal treatment at 60 °C.

For the doped samples, Ag precursor solutions at 3% and 5% (*w*/*w* relative to zinc) were prepared. In this step, 10 mL of the extract was mixed with the corresponding amount of AgNO_3_ for 30 min.

The Ag solution was then added dropwise to the zinc solution, and the mixture was stirred for an additional 60 min. The samples underwent thermal treatment overnight at 60 °C and were subsequently calcined at 400 °C for 60 min. Finally, the powders were ground and labelled as ZnO, 3AgZnO, and 5AgZnO, where 3AgZnO and 5AgZnO denote ZnO doped with 3% and 5% Ag, respectively.

### 2.4. Characterization

To evaluate the structural, physical, and optical properties of the materials, several characterization techniques were performed. FT-IR was used to identify the functional groups present in the synthesized material as well as in the extract, employing a Perkin Elmer spectrophotometer (PerkinElmer, Inc., Waltham, MA, USA) in the range of 400 cm^−1^ to 4000 cm^−1^.

X-ray diffraction was used to determine the crystalline structure and crystallite size of the material. A Bruker D2-PHASER diffractometer (Bruker Corporation, Madison, WI, USA) with Cu Kα radiation (λ = 0.15418 nm), scanning over a 2θ range of 10–80°, was employed.

CL measurements were performed on a Gatan mono-CL4 system (Gatan, Inc., Pleasanton, CA, USA) at 300 K with a photomultiplier sensor, sensitive in the spectrum range from 200 nm to 900 nm, mounted on the SEM system (Thermo Fisher Scientific, Hillsboro, OR, USA).

Scanning electron microscopy was used to observe the morphology of the samples, while energy-dispersive X-ray spectroscopy (Thermo Fisher Scientific, Hillsboro, OR, USA) was employed to confirm the elements present in the synthesized nanoparticle samples. SEM analysis was performed using a JEOL FIB-4500 SEM (JEOL Ltd., Tokyo, Japan) at 15 kV. Chemical composition was evaluated by EDS with an Oxford Instruments detector mounted on the SEM system (Oxford Instruments NanoAnalysis, High Wycombe, UK).

XPS measurements were carried out using a SPECS XPS system equipped with a PHOIBOS 150 WAL hemispherical analyzer and a monochromatic Al Kα X-ray source (1486.6 eV, 200 W) (SPECS Surface Nano Analysis GmbH (SPECS GmbH), Berlin, Germany). High-resolution spectra were acquired at a pass energy of 20 eV under ultrahigh vacuum conditions (<1 × 10^−9^ Torr).

### 2.5. Photocatalytic Activity

The degradation was carried out using a 15 ppm MB solution. The dye solution, 50 mL, was mixed with 50 mg of the nanoparticles and stirred for 30 min in the dark.

The solution was then transferred into a reactor equipped with ultraviolet lamps. Aliquots were collected every 10 min during the first hour, and subsequently every 30 min until completing a total of 180 min.

The dye concentration was measured the following day using a PerkinElmer UV–Vis spectrophotometer (Lambda 365) (PerkinElmer, Inc., Waltham, MA, USA) in the range of 400 nm to 800 nm.

## 3. Results and Discussion

### 3.1. FT-IR Spectroscopy

**Figure 1:**
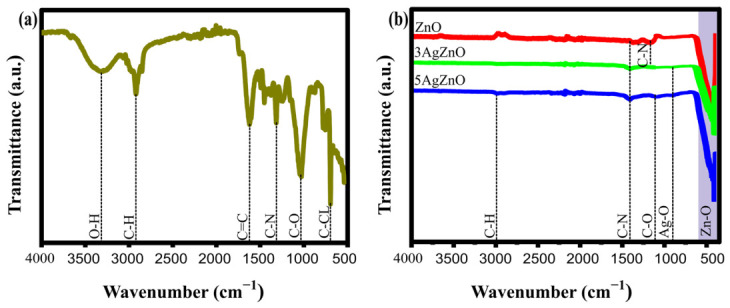
(**a**) FT-IR spectrum of *Cylindropuntia cholla* and (**b**) ZnO and Ag-doped ZnO nanoparticles.

Figure 1a,b show the spectra of the *Cylindropuntia cholla* root and ZnO nanoparticles, respectively. The samples were analyzed in the range of 400 cm^−1^ to 4000 cm^−1^.

The *Cylindropuntia cholla* root extract contains various compounds, including carboxylic acids, alcohols, ethers, esters, aldehydes, and aromatic compounds [30]. FT-IR analysis confirmed the presence of these functional groups through characteristic absorption bands at 3306 cm^−1^ (O–H stretching of alcohols), 2909 cm^−1^ (C–H stretching of aldehydes), 1627 cm^−1^ (C=C stretching of aromatic compounds), 1343 cm^−1^ (C–N stretching of amines), 1025 cm^−1^ (C–O stretching of alcohols, ethers, and esters), and 707 cm^−1^ (C–Cl). These results are consistent with the components reported for the extract [33,34,35].

The nanoparticles show peaks between 400 cm^−1^ and 406 cm^−1^, attributed to the Zn–O bond, another peak at 1378 cm^−1^ corresponding to C–N, and 1059 cm^−1^ due to C=O. In the case of the 3AgZnO and 5AgZnO samples, there is a peak between 860 and 880 cm^−1^ that can be attributed to the interaction between Ag–O [28]. The FT-IR spectrum confirms the formation of ZnO and ZnO with the presence of Ag. The other functional groups present in the nanoparticles can be attributed to the extract (C–H, C–N, and C=O) [36]. These functional groups, particularly hydroxyl (–OH), are known to play a key role in the reduction of metal ions and stabilization of nanoparticles [37,38]. Although the specific contribution of each functional group was not individually verified, the FT-IR results, together with previous reports, support their role in the green synthesis of Ag-doped ZnO nanoparticles.

### 3.2. Possible Formation Mechanism of Ag-Doped ZnO Nanoparticles

Green synthesis of nanoparticles consists of three main phases: reduction, nucleation, and stabilization [39]. Metal ions are reduced to their elemental form due to the interaction between the bioactive compounds in the extract and the metal salt. The metal atoms organize to form a nucleus, creating clusters of atoms [40]. The compounds from the extract adhere to the surface, aiding growth and preventing nanoparticle agglomeration.

**Figure 2:**
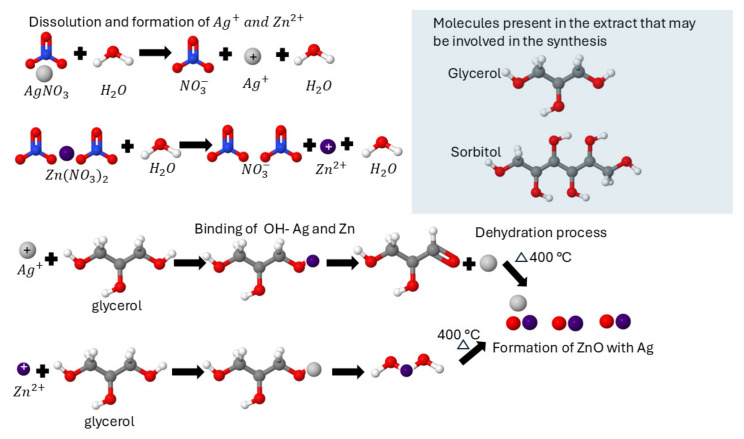
Systematic representation of the possible mechanism for the formation of Ag-doped ZnO nanoparticles.

Figure 2 shows a possible mechanism for nanoparticle growth, which may be associated with the presence of compounds such as glucitol or glycerol, compounds that are abundant in the root of *Cylindropuntia cholla*.

Dissolution of the precursors and formation of Ag^+^ and Zn^2+^ ions (1)–(2) [41,42,43]:

(1) $$AgNO_{3}\left(aq\right)\to Ag^{+}\left(aq\right)+NO_{3}^{-}\left(aq\right)\;\;\;\;\;$$

(2) $$Zn(NO_{3}{)}_{2}\cdot 6H_{2}O\;\left(s\right)\to {Zn}^{+2}\left(aq\right)+2NO_{3}^{-}\left(aq\right)+\;{6H}_{2}O(l)$$

The hydroxyl groups (–OH) of the phenolic compounds in the extract bind to Zn^2+^ and Ag^+^ ions (3)–(4) [44,45,46]:

(3) $${Zn}^{+2}(aq)+2OH^{-}(aq)\to Zn(OH{)}_{2}(s)$$

(4) $$Ag^{+}(aq)+OH^{-}(aq)\to AgOH(s)$$

Upon applying heat, a dehydration process occurs, producing ZnO, while Ag^+^ is incorporated into the lattice or deposited on the ZnO surface (5) [47,48]:

(5) $$2AgZn(OH{)}_{3}(s)\overset{∆}{\to }2AgZnO(s)+{3H}_{2}O(g)$$

### 3.3. X-Ray Diffraction (XRD)

**Figure 3:**
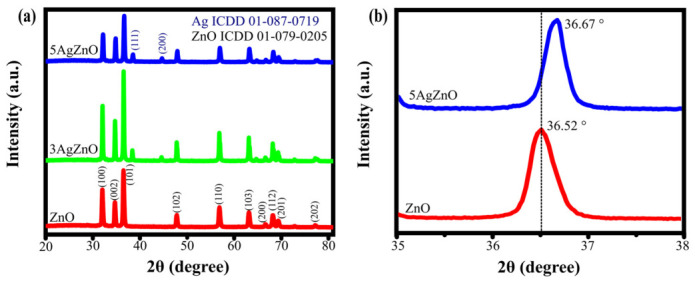
(**a**) XRD patterns ZnO, 3AgZnO and 5AgZnO, and (**b**) shift towards a higher angle.

Figure 3a shows the diffraction pattern of the synthesized nanoparticles. The 2θ was measured from 10° to 80° with a step size of 0.02°. Peaks are observed at 32.04°, 34.71°, 36.45°, 47.86°, 56.85°, 63.17°, 66.70°, 68.17°, 69.39°, and 77.24°, corresponding to the (100), (002), (101), (102), (110), (103), (200), (112), (201), and (202) planes, respectively, which correspond to the hexagonal wurtzite crystalline structure of ZnO according to the crystallographic card ICDD 01-087-0719 [49,50]. The doped samples show the same peaks corresponding to ZnO, as well as additional ones at 38.43°, 44.61°, and 64.70°, whose angles correspond to the (111), (200), and (220) planes. These planes can be attributed to Ag according to the crystallographic card ICDD 01-079-0205 [51]. The crystallite size was calculated using Scherrer’s Equation (6) [52]:

(6) $$D_{hkl}=\frac{k\lambda }{\beta_{d}cos\theta }$$

where

D = Crystallite size (nm)

k = Constant (0.9)

β = Full width at half maximum (FWHM) of the most intense peak

λ = X-ray wavelength (0.154 nm)

θ = Bragg angle (rad)

Figure 3b shows a slight shift to higher angles in the ZnO peak, from 36.52° to 36.67° for the 5AgZnO sample. Since Ag has a larger ionic radius than Zn, the introduction of Ag^+^ into the ZnO system can induce distortions in the crystalline structure, which are reflected in both the peak shift and the increase in crystallite size [53,54]. The difference in ionic radii between Ag^+^ (126 pm) and Zn^2+^ (74 pm) also favors the formation of metallic Ag on the surface rather than full substitution into the lattice [55].

Previous studies have reported that the incorporation of Ag into the ZnO lattice is not straightforward due to this mismatch in ionic radii [56,57]. For example, Hussain et al. (2024) observed an increase in crystallite size after Ag doping, which they attributed to the presence of Ag mainly on the surface [58]. Similarly, Al-Bataineh et al. (2022) reported a decrease in crystallinity along with an increase in crystallite size, as well as a shift toward higher diffraction angles, which they related to lattice stress induced by the size difference between Zn and Ag [59].

These results suggest that Ag is not fully incorporated into the ZnO lattice. Instead, due to the difference in ionic radii between Ag^+^ and Zn^2+^, Ag incorporation is likely limited, favoring the formation of metallic Ag on the surface or at the interface of ZnO. Therefore, the synthesized material can be considered as partially doped ZnO with the possible presence of surface-deposited Ag.

### 3.4. Cathodoluminescence

**Figure 4:**
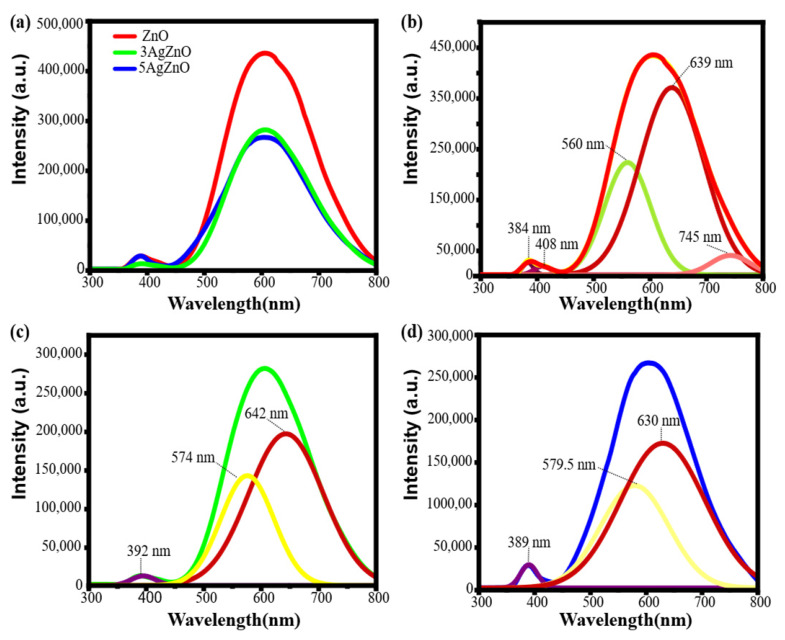
CL (**a**) ZnO, 3AgZnO and 5AgZnO, (**b**) ZnO, (**c**) 3AgZnO and (**d**) 5AgZnO.

Figure 4a shows the cathodoluminescence spectrum of the three samples. A decrease in the emission intensity is observed with the addition of Ag; this reduction can be attributed to the decreased recombination rate of electron–hole pairs, that is, the lifetime of these charge carriers is extended [60,61]. 

Figure 4b,c present the deconvoluted CL spectra, where two emission peaks can be distinguished. For pure ZnO, the Near Band Edge (NBE) emissions is observed at 384 nm, while the introduction of Ag shifts this peak slightly to 392 nm and 389 nm for 3AgZnO and 5AgZnO [62]. In addition to the NBE band, ZnO exhibits several deep level emission (DLE) peaks at 408, 560, 639, and 745 nm, which are associated with intrinsic defect states [63,64].

After Ag incorporation, the emission changes notably. The 3AgZnO sample displays peaks at 574 nm and 642 nm, while 5AgZnO shows peaks at 579.5 nm and 630 nm which fall in the yellow–green and near-infrared regions and can be associated with deep level defects including oxygen vacancies, oxygen interstitials, zinc interstitials and defect complexes [65,66,67].

### 3.5. X-Ray Photoelectron Spectroscopy

**Figure 5:**
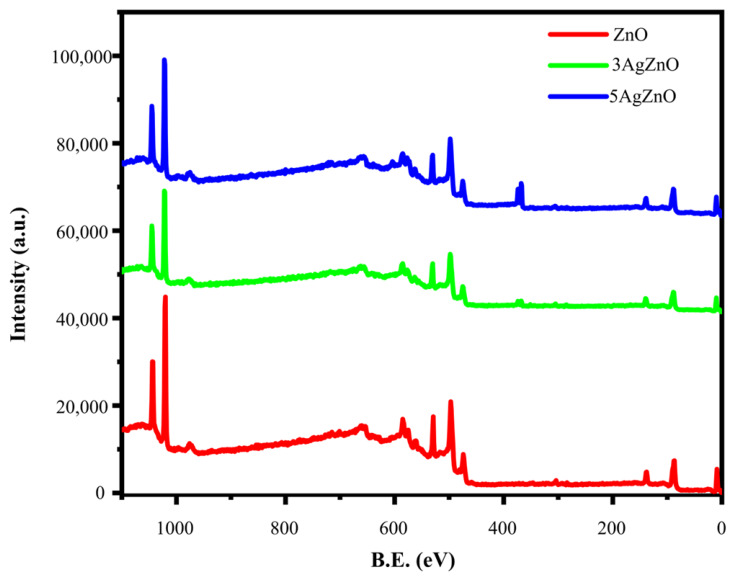
XPS spectrum of ZnO, 3AgZnO and 5AgZnO.

Figure 5 shows the full XPS spectrum of the nanomaterials, where binding energies corresponding to Zn, O, and Ag can be observed in the doped sample.

**Figure 6:**
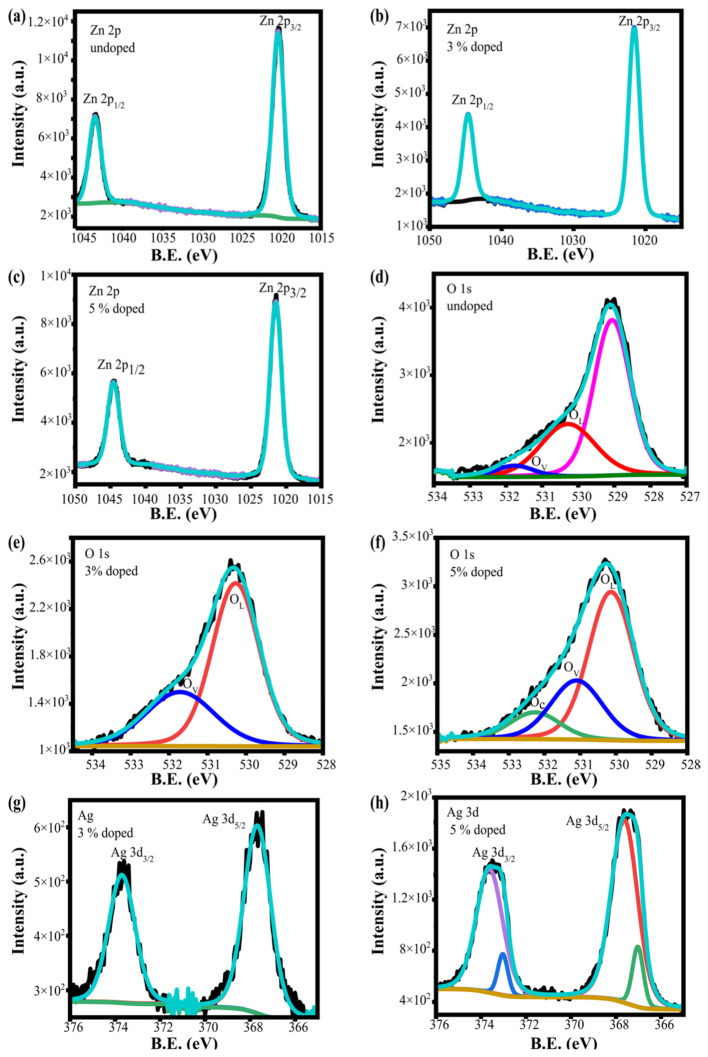
High-resolution XPS spectra of Zn 2p for (**a**) ZnO, (**b**) 3AgZnO, and (**c**) 5AgZnO; O 1s for (**d**) ZnO, (**e**) 3AgZnO, and (**f**) 5AgZnO; and Ag 3d for (**g**) 3AgZnO and (**h**) 5AgZnO. The black line corresponds to the data without prior treatment, while the cyan line represents the data after treatment. In the case of oxygen, the red color corresponds to lattice oxygen, blue to oxygen vacancies, and green to chemisorbed oxygen.

Figure 6 presents the high-resolution XPS spectra. Figure 6a–c display the Zn 2p region, with binding energies of 1020.37 eV and 1043.48 eV for ZnO, 1021.56 eV and 1044.67 eV for 3AgZnO, and 1021.46 eV and 1044.56 eV for 5AgZnO, all showing a spin–orbit splitting of 23.11 eV [68,69]. A shift toward higher binding energies of 1.19 eV and 1.08 eV is observed upon Ag doping, suggesting a slight loss of electronic density around Zn due to the incorporation of Ag atoms [70].

Figure 6d–f correspond to the O 1s spectra, showing binding energies of 529.06 eV, 530.29 eV, and 531.76 eV for ZnO; 530.27 eV and 531.72 eV for 3AgZnO; and 530.13 eV, 532.27 eV, and 531.10 eV for 5AgZnO. According to the literature, peaks around 530 ± 0.5 eV are assigned to lattice oxygen (O_L_) in the Zn–O bond, those near 531.5 eV to oxygen vacancies (O_v_), and those at 532.5 ± 0.5 eV to chemisorbed oxygen (O_c_). Therefore, besides lattice oxygen, the spectra reveal the presence of oxygen vacancies and chemisorbed species [60,71].

For Ag, Figure 6g,h show binding energies of 367.67 eV and 373.70 eV for 3AgZnO, and 367.63 eV, 373.03 eV, 367.03 eV, and 373.62 eV for 5AgZnO. Reported values of 367.7 eV and 373.6 eV correspond to Ag^+^ and Ag^0^, respectively, indicating the coexistence of both oxidation states in the doped samples [72,73,74,75].

### 3.6. SEM and EDS

**Figure 7:**
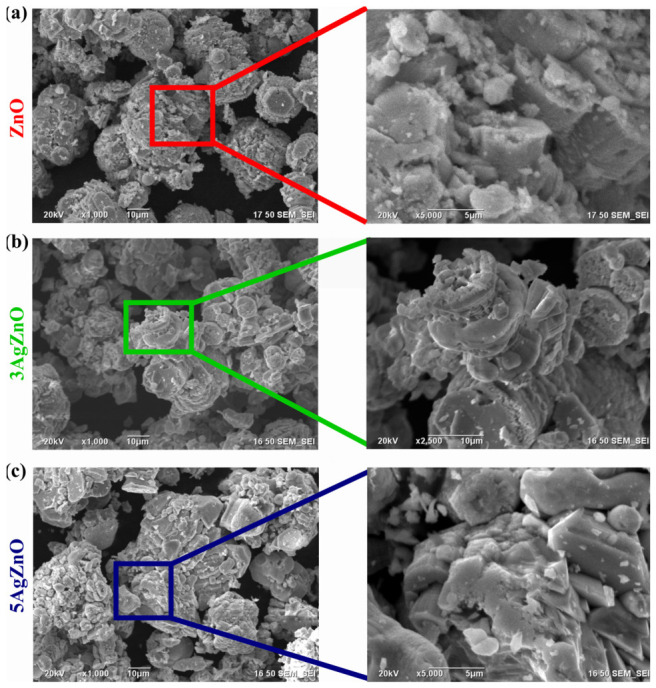
SEM (**a**) ZnO, (**b**) 3AgZnO and (**c**) 5AgZnO.

Figure 7 shows the micrographs of the nanoparticles. Figure 7a corresponds to the ZnO material, where hexagonal shapes can be observed, along with agglomerates of particles that do not have a homogeneous shape. In Figure 7b, corresponding to 3AgZnO, agglomerates with cylindrical formations can be observed, as if the material were growing or grouping into columns. Hexagons can be seen but are less defined compared to ZnO. In the case of 5AgZnO, larger agglomerates can be observed, with poorly defined shapes and no particular order. The samples do not exhibit a homogeneous shape or distribution; however, a change in morphology is observed when ZnO is doped with Ag. Upon the addition of Ag, brighter areas are also observed, which may indicate an increase in density.

**Figure 8:**
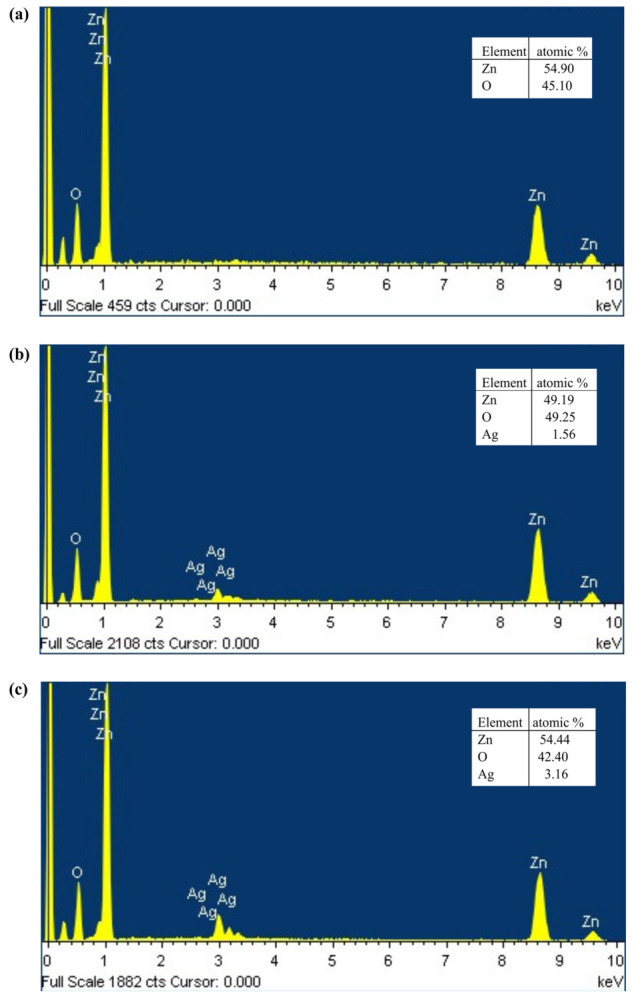
EDS (**a**) ZnO, (**b**) 3AgZnO and (**c**) 5AgZnO.

Figure 8 shows the results obtained by EDS. Oxygen and zinc were detected in all the samples. In the case of the doped samples, Ag was also detected. The Ag content increased from 1.56% in the 3AgZnO sample to 3.16% in 5AgZnO, which is consistent with the methodology performed. This demonstrates the addition of small amounts of silver into the zinc oxide.

### 3.7. Methylene Blue Degradation Test

**Figure 9:**
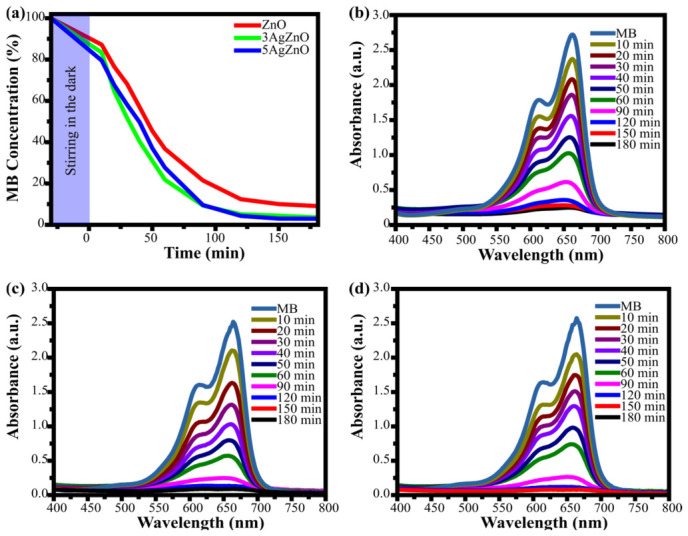
(**a**) Photocatalytic activity of ZnO nanoparticles and Ag-doped ZnO nanoparticles for the degradation of MB under UV light. UV-Vis spectrum of (**b**) ZnO, (**c**) 3AgZnO and (**d**) 5AgZnO for the degradation of MB under UV light.

Figure 9a shows the degradation after 30 min of stirring in the dark, followed by 180 min under UV light irradiation, illustrating the photocatalytic degradation of MB. The graph shows how the doped material degrades a greater percentage of the dye and that, in half the time, it has already degraded what ZnO takes 180 min to achieve. The 5AgZnO sample exhibited the highest degradation efficiency.

Figure 9b shows the result of measuring the absorbance of water mixed with the ZnO catalyst and the dye. A maximum absorption peak corresponding to 664 nm, characteristic of methylene blue, is observed. As time progresses, a decrease in the intensity of the peaks is seen; in the first 10 min, there is a 12.8% decrease, at 90 min it decreases by 78.5%, and at the final time, it has decreased by 90.9%. This indicates the degradation of the dye, as the characteristic blue colour fades over time.

Figure 9c corresponds to the nanoparticles doped at 3% (3AgZnO). In this spectrum, the maximum absorption peak is again at 664 nm, corresponding to methylene blue. As time progresses, the intensity decreases from 16.6% to 96.4% from 10 min to 180 min, respectively. The reduction in the presence of the dye is greater from the beginning. This increase in the degraded percentage represents a significant improvement in photocatalytic activity. In the case of 5AgZnO, the spectrum again shows the absorption peak at 664 nm (Figure 9d). At 10 min, degradation is 20.4%, and at 90 min, it has already degraded 90.4%, a value close to that achieved by ZnO but 90 min later. This means that the nanoparticles doped at 5% managed to degrade in half the time what ZnO without Ag presence achieved.

The highest degradation was achieved using 5AgZnO. The Ag doping in the ZnO nanoparticles increased the photocatalytic activity from 90.9% to 97.0% at 180 min, confirming that doping indeed improves the photocatalytic activity of ZnO. The synthesized nanoparticles were compared with previously reported Ag–ZnO systems, as summarized in Table 1. The results indicate that the degradation efficiency achieved in this work is comparable to those reported in the literature, even under conditions involving higher initial MB concentrations. Moreover, previous studies have shown that excessive Ag content can lead to a decrease in efficiency. This may be because an excess of dopant deforms the crystal lattice or that agglomeration favors the recombination of the electron–hole pair. In contrast, the present study demonstrates that relatively low Ag content is sufficient to achieve significant improvement, highlighting the effectiveness of the proposed synthesis approach.

Kwon and Kim (2020) doped ZnO with 0.3%, 0.5%, 0.4%, 0.5%, 0.2%, and 0.1% Ag [78]. The band gap decreased from 3.3 eV to 3.25 eV; their optimal percentage was 0.1%, which also presented the lowest band gap value. It degraded 92.4% of MB, while pure ZnO degraded 61.8%, significantly improving photocatalytic activity. They performed XPS analysis and found AgO and Ag_2_O; there was no presence of metallic silver (Ag^0^), demonstrating the formation of Ag–O bonds. Due to oxygen vacancies, the separation of the electron–hole pair was improved.

Ersöz and Altintas Yildirim (2022) doped ZnO with Ag at 1%, 2%, 3%, and 4% [83]. A decrease in the band gap from 3.13 eV to 2.75 eV was observed for ZnO doped with the highest percentage. The degradation of methylene blue improved from 96.78% to 98.66%. It was consistent that the most efficient catalyst is the one with the lowest band gap value. They characterized their samples by photoluminescence, observing a decrease in the maximum UV emission intensity, which suggests a reduction in the recombination rate of charge carriers and, consequently, an improvement in electron–hole separation, to which they attribute the enhanced degradation.

The results obtained are consistent with those previously reported in the literature. With small amounts of Ag, the catalyst efficiency is significantly improved; it is not necessary to add large amounts that increase the cost. In fact, it is evident that adding excessive amounts of Ag reduces the photocatalytic activity.

Photocatalytic activity was modelled using pseudo-first-order kinetics [84,85]. The reaction rate is expressed as (7):

(7) $$-\ln(\frac{C_{t}}{C_{0}})=kt$$

where

*k* = pseudo first order rate constant

C_0_ = Initial dye concentration

C_t_ = dye concentration at a specific time

t = time 

**Figure 10:**
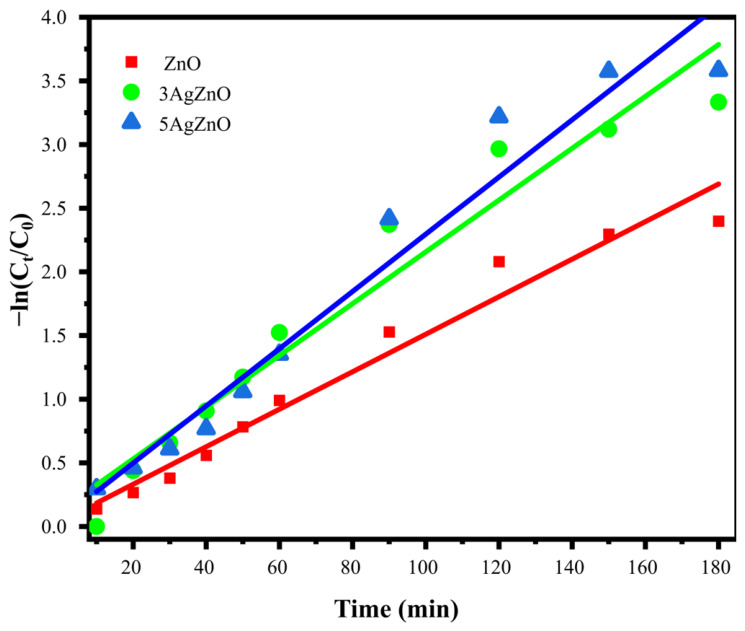
Kinetics of MB degradation.

The result of plotting −ln($\frac{C_{t}}{C_{0}}$) versus time (Figure 10) is used to calculate the constant *k*. The *k* values are 0.01474 min^−1^, 0.02034 min^−1^, and 0.02247 min^−1^ for ZnO, 3AgZnO, and 5AgZnO, respectively. 5AgZnO shows a slight improvement compared to 3AgZnO, but both are faster than undoped ZnO. 

### 3.8. Methylene Blue Degradation Mechanism

**Figure 11:**
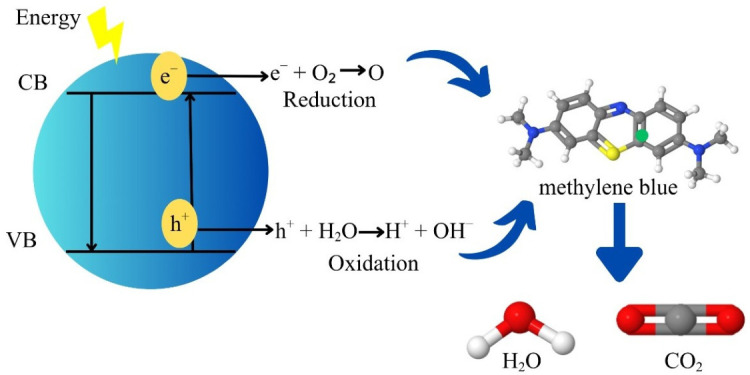
Possible mechanism for the degradation of MB.

The nanoparticles promote electrons from the valence band to the conduction band, producing an electron–hole pair that forms highly oxidizing and reducing radicals, which interact with the methylene blue molecule, producing H_2_O_2_, CO_2_, and molecular residues, as shown in Figure 11 [86,87]. When the nanoparticles are irradiated with an energy source equal to or greater than their band gap, electrons in the valence band (VB) of ZnO absorb energy to jump to the conduction band (CB), generating an electron–hole pair (e^−^/h^+^) [88].

(8) $$AgZnO+hv\;\to \;e_{CB}^{-}+\;h_{VB}^{+}$$

Electrons in the conduction band (e^−^) react with molecular oxygen (O_2_) in the medium to form superoxide radicals ($O_{2}^{•-}$):

(9) $$O_{2}+\;e_{BC}^{-}\to O_{2}^{•-}$$

The hole (h^+^) in the valence band reacts with water molecules (H_2_O) adsorbed on the ZnO surface and hydroxide ions (OH^−^) to form hydroxyl radicals (OH•) [89]:

(10) $$H_{2}O+h_{VB}^{+}\;\to H^{+}+OH•$$

(11) $$h_{VB}^{+}+OH^{-}\;\to OH•$$

The reactive oxygen species (ROS) react with MB, breaking the bonds of its organic structure and generating simpler intermediate compounds, as well as H_2_O_2_ and CO_2_ [90,91]:

(12) $${(O}_{2}^{•-},\;OH•)+MB\;\to \;H_{2}O_{2}+CO_{2}+degraded\;products$$

The degradation of MB can be divided into four stages. First, demethylation hydroxyl radicals (•OH) attack the diethylamino groups of MB, progressively removing methyl groups (–CH_3_) to form intermediates such as Azure B, Azure A, Azure C, and finally thionine. Second, the cleavage of aromatic rings occurs; •OH radicals attack the central aromatic ring of MB particularly the one containing sulphur, followed by the rupture of the side aromatic rings, resulting in smaller aromatic fragments.

In the third stage, these fragments are oxidized to intermediate organic species, including R–NH_3_^+^, phenol, aniline, and aldehydic or carboxylate compounds. Finally, during the fourth stage, these intermediates undergo complete mineralization to yield simple, non-toxic inorganic products such as CO_2_, H_2_O, SO_4_^2−^, and NH_4_^+^. The degradation process is initiated by the attack of hydroxyl radicals on the C–S^+^=C functional group, which represents the most reactive site in the MB structure and triggers subsequent bond cleavage and oxidation reactions [10,92].

## 4. Conclusions

ZnO and Ag-doped ZnO nanoparticles were successfully synthesized using a green synthesis approach with *Cylindropuntia cholla* root extract. The biomolecules present in the extract acted as reducing and stabilizing agents. These results demonstrate the potential of this plant as a reducing and stabilizing agent for sustainable synthesis, eliminating the need for additional chemical reducing or stabilizing agents. Characterization confirmed the formation of hexagonal wurtzite ZnO nanoparticles with average crystallite sizes between 23.46 nm and 32.53 nm. FT-IR analysis identified hydroxyl, carbonyl, and amine groups in the extract, which played a role in nanoparticle stabilization. XRD and XPS confirm the presence of Ag within the sample. The presence of metallic Ag detected by XRD suggests that some of the dopant is not fully incorporated into the ZnO network. Furthermore, the lack of advanced characterization techniques, such as TEM or elemental mapping, limits a detailed understanding of the dopant distribution at the nanoscale.

Photocatalytic degradation tests demonstrated that Ag-doped ZnO exhibited superior activity compared to pure ZnO. The photocatalytic activity increased from 90.9% to 97.0% under UV light upon doping ZnO with 5% Ag, highlighting the beneficial effect of Ag incorporation. These results demonstrate that the incorporation of Ag through a green route is an effective strategy to enhance the photocatalytic activity of ZnO. Future studies could focus on evaluating performance under visible light and the reuse of the photocatalyst in real systems and employing advanced characterization techniques to better understand the structure.

## Figures and Tables

**Table 1:** Comparison with previous works on methylene blue degradation.

Material	Time	Degradation	Dye Dose	Catalyst Dose	Ref.
ZnO	100 min	94.3%	10 mg/L	1 g	[76]
3%Ag-doped ZnO	100 min	96.5%			
5%Ag-doped ZnO	100 min	97.5%			
ZnO	240 min	56%	5 ppm	0.15 g	[77]
0.5%Ag-doped ZnO	240 min	58%			
1%Ag-doped ZnO	240 min	83%			
2%Ag-doped ZnO	240 min	92%			
5%Ag-doped ZnO	240 min	62%			
10%Ag-doped ZnO	240 min	60%			
30%Ag-doped	240 min	55%			
ZnO	210 min	61.8%	10 ppm	50 mg	[78]
0.5%Ag-doped ZnO	210 min	82.6%			
0.1%Ag-doped ZnO	210 min	92%			
0.2%Ag-doped ZnO	210 min	89.9%			
Ag-ZnO (composite)	90 min	91%	1 mg/50 mL	20 mg/L	[79]
ZnO	240 min	65.6%	2 × 10^−5^ M	0.1 mg/L	[80]
3%Ag-doped ZnO	240 min	82.6%			
5%Ag-doped ZnO	240 min	81.2%			
ZnO	60 min	53%	1 mg/L	0.079 mg/L	[81]
Ag-doped ZnO	30 min	95%			
ZnO	120 min	69%	10 ppm	100 mg/100 mL	[82]
7%Ag-doped ZnO	120 min	73%			
ZnO	180 min	90.9%	15 ppm	50 mg	Present work
3AgZnO	180 min	96.4%			
5AgZnO	180 min	97.0%			

## Data Availability

The original contributions presented in this study are included in the article. Further inquiries can be directed to the corresponding authors.
